# National audit on the appropriateness of CT and MRI examinations in Luxembourg

**DOI:** 10.1186/s13244-019-0731-9

**Published:** 2019-05-20

**Authors:** Aurélien Bouëtté, Alexandra Karoussou-Schreiner, Hubert Ducou Le Pointe, Martijn Grieten, Eric de Kerviler, Léon Rausin, Jean-Christophe Bouëtté, Patrick Majerus

**Affiliations:** 1 Radiation Protection Department, Health Directorate, Ministry of Health, Allée Marconi - Villa Louvigny, Luxembourg, L-2120 Luxembourg; 2 Department of Pediatric Imaging, Armand Trousseau Hospital, APHP, The MAMUTH Hospital (University Department for Innovative Therapies in Musculoskeletal Diseases), Sorbonne Université, Paris, France; 3 Radiology Department, Ziekenhuis Oost-Limpurg, Schiepse bos 6, 3600 Genk, Belgium; 4 Service de Radiologie, Hôpital Saint-Louis, Assistance Publique des Hôpitaux de Paris, 1 Avenue Claude Vellefaux, 75010 Paris, France; 5 Service d’Imagerie Médicale, Centre Hospitalier Régional de la Citadelle, Liège, 1 Boulevard du 12ème de Ligne, 4000 Liège, Belgium; 6 Statistic, Independent Statistician, Montréal, QC Canada

**Keywords:** Clinical audit, Referral, Guidelines, Computed tomography scanner, Magnetic resonance imaging

## Abstract

**Objectives:**

In Luxembourg, the frequency of CT and MRI examinations per inhabitant is among the highest in Europe. A national audit was conducted to evaluate the appropriateness of CT and MRI examinations according to the national referral guidelines for medical imaging.

**Methods:**

Three hundred and eighty-eight CT and 330 MRI requests corresponding to already performed examinations were provided by all radiology departments in Luxembourg. Four external radiologists evaluated the clinical elements for justification present in each request. They consensually assessed the appropriateness of each requested examination with regard to the national referral guidelines and their clinical experience.

**Results:**

The appropriateness rate (AR) was higher for MRI requests than for CT requests (79% vs. 61%; *p* < 0.001). AR was higher for requests referred by medical specialists rather than by general practitioners, both for CT requests (70% vs. 37%; *p* < 0.001) and MRI requests (83% vs. 64%; *p* = 0.002). For CT, AR was higher when the requests concerned paediatric rather than adult patients (82% vs. 58%; *p* < 0.001), when the radiology departments were equipped with both CT and MRI units rather than with only CT units (65% vs. 47%, *p* = 0.004) and when the requests concerned head-neck (79%), chest (77%) and chest-abdominal-pelvic (81%) areas rather than spinal (28%), extremity (51%) and abdominal-pelvic (63%) areas (*p* < 0.001).

**Conclusions:**

The appropriateness of CT and MRI in Luxembourg is not satisfactory and collective efforts to improve should be continued. The focus should be on general practitioners and on spinal CT examinations.

**Electronic supplementary material:**

The online version of this article (10.1186/s13244-019-0731-9) contains supplementary material, which is available to authorized users.

## Key points

- A high proportion of CT requests (39%) and MRI (21%) requests are inappropriate.
- Overall, requests from general practitioners are less appropriate that those from medical specialists.
- Requests concerning spinal CT examinations are less appropriate than the others.
- The appropriateness is better for CT requests concerning children than adults.
- The appropriateness is better for CT requests in the radiology departments equipped with both CT and MRI units than in those equipped with only CT units.

## Introduction

There is a growing focus on the implementation of the principle of justification of medical exposures in Europe, promulgated by the European Commission (EC) [1], the national radiological protection competent authorities [2] and professional societies [3, 4]. In 2007, an International Atomic Energy Agency (IAEA) consultation already showed a significant level of inappropriate use of medical exposures [5]. In 2012, the IAEA together with the World Health Organisation (WHO) launched the “Bonn call for action” of which one of the actions is to enhance the implementation of the principle of justification [6]. In 2017, the Heads of the European Radiological protection Competent Authorities (HERCA) identified an urgent need for improvement and coordinated a European Action Week on the inspection of justification, focussing on radiology departments [7].

Concerned professional societies in Europe recognised a need for further research in the field of justification of medical imaging [8]. A European Society of Radiology (ESR)-led EC survey indicated that referral guidelines were insufficiently known by the health professionals, and suggested that clinical audits should be carried out for monitoring the use and implementation of guidelines [9]. ESR recently identified the appropriateness of requests as a key factor for the evaluation of the quality of work in radiology through its definition of the concept on value-based radiology, and indicated that it could be measured by the analysis of the compliance of requests with imaging referral guidelines [10].

In 2015, a European study revealed a relatively high exposure per inhabitant in Luxembourg, mainly due to the use of CT examinations [11]. The Ministry of Health and the Ministry of Social Security in Luxembourg launched a national action plan composed of four main actions: audits of imaging requests, awareness campaigns for public and health professionals, training of medical referrers and inspection of radiology departments. In 2016, Luxembourg was equipped with 17 CT scanners and 12 MRI units per 1 million inhabitants, and the use of medical imaging per inhabitant was among the highest in Europe, with 211 CT and 83 MRI examinations per 1000 inhabitants [12]. The Radiation Protection Department (RPD) of the Health Ministry in Luxembourg performed two national audits between 2016 and 2017. The first one concerned the adequate completion of medical imaging requests for all types of imaging examinations and revealed that absolutely no clinical elements for justification were indicated in 19% of all requests, despite the fact that it is required by national regulation, but this figure was less than 8% for CT and MRI requests [6]. The goal of this second audit was to go one step further by verifying if the clinical elements for justification indicated on MRI and CT requests were consistent with the appropriateness of the medical imaging examination requested.

## Materials and methods

In 2016, Luxembourg disposed in total of ten radiology departments installed in four regional hospitals, among which seven were equipped with both CT and MRI units, two with only a CT scanner and one with no CT or MRI unit. There was no private radiological practice in Luxembourg.

A group of four external auditors was contracted by the Government of Luxembourg to evaluate, on a representative selection of CT and MRI requests provided by all radiology departments performing these types of examinations, the appropriateness of each request with regard to the national referral guidelines and their clinical experience. The guidance on good use of medical imaging jointly elaborated by the French Society of Radiology and the French Nuclear Medicine Society [13] was recognised since 2001 as the national referral guidelines for Luxembourg. It was hence considered as the standard for this audit.

**Fig. 1:**
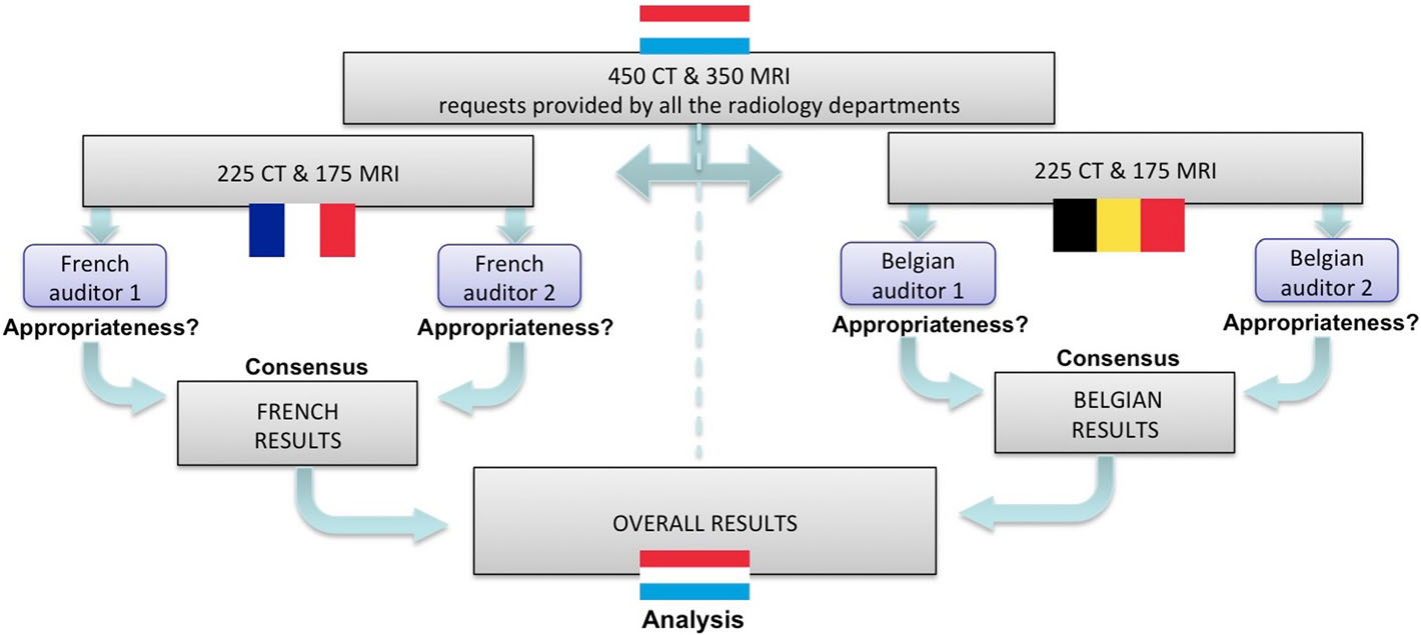
Flowchart showing the methodology of the audit

The audit was coordinated by the members of the RPD (authors 1, 2, 8), who described in a protocol the purpose of the audit, the constitution of the auditor team, the collection of the sample of requests, the evaluation of the appropriateness of each request by the auditors and data analysis. Figure 1 summarises the audit design according to this protocol.

### Constitution of the auditor team

The evaluation of the requests was carried out by a team of auditors composed of one pair of radiologists from France (authors 3, 5) and one pair of radiologists from Belgium (authors 4, 6). It was chosen to include a single specialist in paediatric radiology in each pair in order to have two well-balanced pairs of radiologists with skills in both adult and paediatric radiology. Each radiologist had more than 30 years of experience in CT and MRI imaging and was already familiarised with the guidelines, due to the fact that the guidelines adopted in Luxembourg were the French guidelines and that the guidelines adopted in Belgium were also derived from these guidelines [14].

In addition to the audit protocol, each auditor was provided with a dedicated assessment form and instructions for carrying out the evaluation of the appropriateness of each request. The auditors had to complete the assessment form for each request with data about the patient (gender, age group), referrer (medical speciality), requested examination (imaging modality, type of examination), clinical elements for justification (clinical background, question to be answered by the examination), recommendation in the guidelines (clinical situation found in the guidelines, appropriateness according to its recommendations), conclusion of the auditor (appropriateness according to his evaluation) and, in case of inappropriate requests, complementary information concerning the reason (lack of information, other type of examination instead). The auditors were not informed from which radiology department each request was collected and if it was equipped with an MRI unit or not.

The instructions (see Additional file 1) provided guidance to the auditors on how to complete data in the assessment form. This assessment form was basically an Excel table with one line per request and one column per data to be completed. To help the auditors to find and report the clinical situations of the guidelines, it also included a hypertext link to the referral guidelines web page and drop-down menus listing the section titles and clinical situations of the guidelines. Before the beginning of the evaluation, members of the RPD held a teleconference with all auditors to verify that the audit protocol and instructions for evaluation were well understood and to provide additional clarifications, as appropriate. The auditors were also invited to report in the assessment form any potential difficulties encountered during the evaluation of each request.

### Collection of the sample of requests

Three months prior to the beginning of the audit, the directors of the four regional hospitals in Luxembourg were individually contacted and informed about the audit protocol. Between September and November 2016, members of the RPD (authors 1, 2) visited the heads of department (in Luxembourg the head of department is a radiographer) in each of the nine radiology departments equipped with CT or MRI units. The RPD briefly reminded them of the context and purpose of the audit, and asked to be provided with a sample of 50 CT requests and 50 MRI requests, as appropriate, concerning consecutive examinations already performed in the department during the week of the visit. The members of the RPD were provided with anonymised copies of these requests. The RPD did not consider verifying whether the sample of requests provided by the radiology departments actually corresponded to consecutive examinations performed during the week, or whether it was representative of the typical activity of the departments. They collected all the requests provided by the radiology departments, attributed them reference numbers, masked all the institutional identifiers, mixed them together and separated them into two equal lots with the same number of CT and MRI requests.

### Evaluation of the appropriateness of each request by the auditors

The first lot of requests was evaluated by the French pair of auditors, and the second lot was evaluated by the Belgian pair of auditors. The two radiologists of each pair both received exactly the same sample of requests to evaluate.

Each auditor had to evaluate individually the appropriateness of each request he received, based on the available elements for justification provided on the request, on the recommendation of the guidelines and on his own expertise as a radiologist. Each auditor had to supply his observations and conclusions in the dedicated assessment form and sent it back to the RPD at the latest 3 months after receiving its sample of request.

Each auditor had to actively search in the national guidelines in order to find the recommendation corresponding to the clinical situation described on each request. In the case where recommendations existed for a specific clinical case, they had to be reported and considered by the auditors. Auditors also had to take into account their own ethical and professional judgement, in the case where no recommendation was found in the national guidelines and in the case where they did not agree with the recommendation.

For each request, each auditor had to conclude on whether the examination requested was appropriate or inappropriate. In the latter case, the auditor had to answer complementary questions regarding whether more clinical elements for justification would have been necessary, whether another examination would have been more appropriate and if so which type of examination was more appropriate.

For each lot of requests evaluated by a pair of radiologists, the RPD compared the preliminary conclusions of the two auditors concerning the appropriateness of each request. The requests for which at least one auditor had provided no conclusion were rejected from the data analysis. The requests for which the two auditors had emitted the same preliminary conclusion were validated. If an auditor had considered a request as appropriate, but the other auditor of the pair had considered it as inappropriate, then a consensus search was conducted. For this purpose, the RPD organised a meeting in France with the two French auditors, and another in Belgium with the two Belgian auditors, during which the auditors had to carefully review and discuss each request for which there was disagreement and to provide a common conclusion concerning its appropriateness. In case of persistent disagreement between the two auditors, the request had to be rejected from data analysis.

In this respect, regardless of whether a search for consensus had been preliminarily performed or not, a request was definitely considered as appropriate (resp. inappropriate), when the two radiologists of each pair concluded that it was appropriate (resp. inappropriate).

#### Data analysis

The results from the evaluation of the two half samples of requests by the two pairs of radiologists were merged for a global analysis of the entire sample. Data analysis was carried out by the RPD, based on the information reported by the auditors in the dedicated assessment form, with additional contribution of an independent statistician (author 7).

The appropriateness rate (AR) for a group was defined as the ratio between the number of appropriate requests in the group and the total number of analysed requests in the group. AR was calculated for both CT requests and MRI requests depending on the gender and age group of the patients (< 18 years / ≥ 18 years), on the anatomical area concerned by the requested examination (head-neck/chest/spine/extremities/abdomen-pelvis/chest-abdomen-pelvis), on the specialty of the medical referrer (medical practitioner/general practitioner/dentist) and on the radiology department concerned by the request. For CT requests, AR was also calculated depending on the type of examination and on the availability of MRI units in the radiology departments concerned by the request.

Comparisons of AR values were performed using a two-proportion test (normal approximation) when two groups were compared and using a Chi-square test when more than two groups were compared. A *p* value of less than 0.05 was considered statistically significant. Statistical tests were carried out using Minitab version 18.

Data analysis was essentially descriptive concerning the subsidiary questions for inappropriate requests and the presence and content of recommendations for the clinical case in the guidelines.

For each pair of auditors, inter-observer agreement between the individual responses of each radiologist prior to a search of consensus concerning the appropriateness of requests was assessed using Cohen’s Kappa-test with Landis Koch interpretation of results.

## Results

### Data sample

All radiology departments equipped with a CT scanner (*n* = 9) or a MRI system (*n* = 7) participated in this audit and provided the RPD with 449 CT requests and 349 MRI requests, together with 2 reports of CT and MRI examinations which were not taken into consideration. Then, 21 CT and 11 MRI requests from the French half sample and 39 CT and 7 MRI requests from the Belgian half sample were rejected because at least one of the auditors provided no conclusion. In most of the cases, the auditors reported that they had encountered a difficulty during the evaluation. For 9 CT and 1 MRI requests, the two auditors provided no conclusion and reported that the requested imaging modality was not CT or MRI (*n* = 3) or that the clinical elements for justification were not present in the request (*n* = 6). For 51 CT and 17 MRI requests, one of the auditors provided a conclusion and considered the requests to be appropriate in 68% of cases for CT (35/51) and in 71% of cases for MRI (12/17), while the other auditor did not conclude and indicated that the clinical elements for justification were not present in the request (*n* = 15), not sufficiently comprehensible for evaluation (*n* = 22), in some cases specifying that they were not written in French (*n* = 13).

### Search of consensus

Of the remaining requests, the individual responses of the two French auditors regarding appropriateness were consistent for 81% of the CT (166/204) and 76% of the MRI (124/164) requests they evaluated, and the responses of the two Belgian auditors were consistent for 81% of the CT (149/185) and 85% of the MRI (145/167) requests they evaluated. Prior to consensus, the inter-observer agreement was moderate between the two French radiologists (*k* = 0.54) and between the two Belgian radiologists (*k* = 0.55).

A consensus was found for all of the 78 CT requests and 58 MRI requests for which there was initial disagreement between the auditors concerning the appropriateness, except for one ankle MRI and one cerebral CT request evaluated by the two Belgian radiologists. The ankle MRI request concerned the search for a lesion in a ligament in the context of persistent pain and each auditor had to base his individual conclusion on his own expertise because this clinical situation could not be found in the guidelines: the non-paediatric radiologist considered that it was appropriate to request an MRI while the paediatric radiologist disagreed because he considered that ultrasound was more appropriate. The cerebral CT request concerned a search of metastasis in a patient with pulmonary adenocarcinoma and the auditors based their individual conclusion on two contradictory recommendations they separately found in two distinct sections of the guidelines: one found in the pneumology section of the guidelines that a head CT was recommended in this case, while the other found in the neurology section that an MRI was recommended instead of a CT. These two particular requests were subsequently rejected from further analysis.

Overall, the analysed sample consisted of 388 CT requests (84% of collected) and 330 MRI requests (96% of collected) for which the appropriateness was definitely stated. It included more than 40 CT and 47 MRI requests from each radiology department.

No difference exceeding 3% was found when comparing the relative distribution of the collected and analysed samples of requests depending on patient gender, patient age group, medical specialty, anatomical area and on the presence of an MRI device on the radiology departments. Table 1 presents the distributions for the collected requests and the analysed requests depending on these parameters, together with the number of appropriate requests and corresponding AR values for both CT and MRI requests.

**Table 1:**
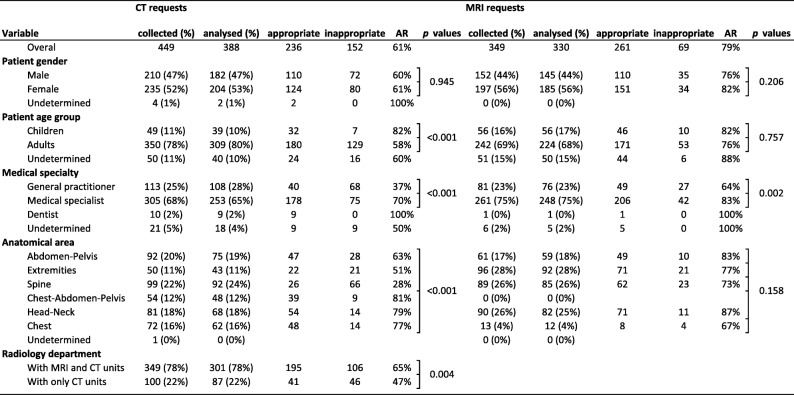
Number and proportion of requests collected in the radiology departments, number and proportion of analysed requests, number of appropriate and inappropriate requests and appropriateness rates (AR) for CT and MRI requests according to patient gender, patient age group, medical speciality of referrer, anatomical area and radiology department depending on the presence of MRI units. Statistical significance if *p* value < 0.05

### Appropriateness rates after consensus

Overall, AR was significantly higher (*p* < 0.001) for MRI requests (79%) than for CT requests (61%). AR was higher (*p* < 0.001) for CT requests referred by medical specialists (70%) than by general practitioners (37%), and the same tendency was also observed for MRI (83% vs. 64%; *p* = 0.002). AR was significantly higher (*p* < 0.001) for CT requests concerning children (82%) than adults (58%), while no such variation was found for MRI requests.

**Fig. 2:**
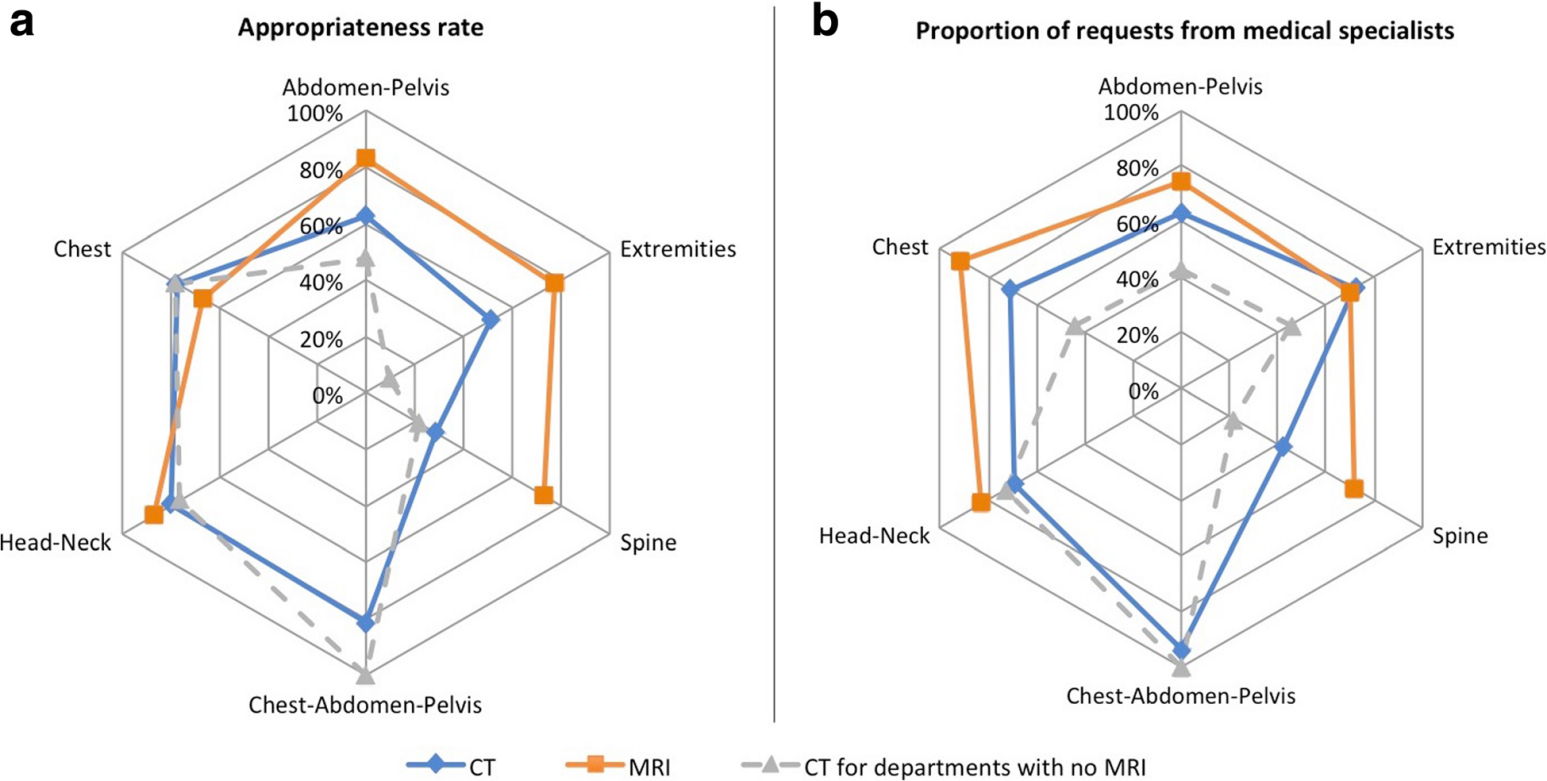
**a** Radar chart showing AR for each anatomical area for CT requests, MRI requests and CT requests for the two radiology departments not equipped with MRI units. **b** Radar chart showing for each anatomical area the proportion of requests from medical specialists depending on each anatomical area for CT request, MRI requests, and for CT requests for the two radiology departments not equipped with MRI units

Figure 2 presents AR according to anatomical area for CT and MRI requests and the proportion of requests from medical specialists. AR was better for CT requests concerning head-neck (79%), chest (77%) and chest-abdominal-pelvic (81%) areas than spinal (28%), extremity (51%) and abdominal-pelvic (63%) areas (*p* < 0.001). No such variation of AR depending on the anatomical area was found for MRI requests. Only 42% of spinal CT requests and 63% of abdominal-pelvic CT requests were from medical specialists, compared to 94% for thorax-abdominal-pelvic CT requests.

Table 2 presents in further details the results for CT depending on the type of examination that was requested for each anatomical area. Requests for lumbar spine CT were the most prevalent inappropriate requests, with 84% of them being inappropriate (51/61), in most cases (46/51) because there was indication of back pain but the auditors agreed with the guidelines that recommended a CT only for a particular clinical condition (21/46) or after another type of examination (25/46) which was not mentioned in the request. Ninety-one percent of the requests for knee CT arthrography were inappropriate (10/11), in most cases (7/10) because the auditors agreed with the guidelines that recommended an MRI examination for the described clinical situation. All of the inappropriate requests on the abdominal-pelvic area were simply reported as abdomen-pelvis CT examination (28/28) and were associated with various clinical situations concerning in most cases the digestive system (16/28) or the urogenital system (7/28).

**Table 2:** Number of appropriate and inappropriate requests and appropriateness rates (AR) for CT according to the type of CT examination

	Appropriate	Inappropriate	AR
Abdominal-pelvic area	47	28	63%
Abdomen-pelvis CT	39	26	60%
Renal CT angiography	2	0	100%
CT-guided abdominal drainage	2	0	100%
CT Urography	4	2	67%
Extremity area	22	21	51%
Knee CT arthrography	1	10	9%
Lower extremities CT (other)	5	5	50%
Lower extremities CT angiography	5	0	100%
Upper extremities CT	6	2	75%
Upper extremities CT angiography	5	4	56%
Spinal area	26	66	28%
Lumbar spine CT	10	51	16%
Cervical spine CT	13	13	50%
Dorsal spine CT	1	2	33%
Spinal CT infiltration	2	0	100%
Chest-Abdominal-Pelvic area	39	9	81%
Chest-abdomen-pelvis CT	32	8	80%
Head-chest-abdomen-pelvis CT	7	1	88%
Head-Neck area	54	14	79%
Cerebral CT	18	9	67%
Brain or Neck Angio-CT	3	2	60%
Temporal bone CT	4	0	100%
Sinus CT	13	3	81%
ORL CT (other)	7	0	100%
Dental CT	9	0	100%
Chest area	48	14	77%
Chest CT	37	13	74%
Chest-abdomen CT angiography	11	1	92%

**Fig. 3:**
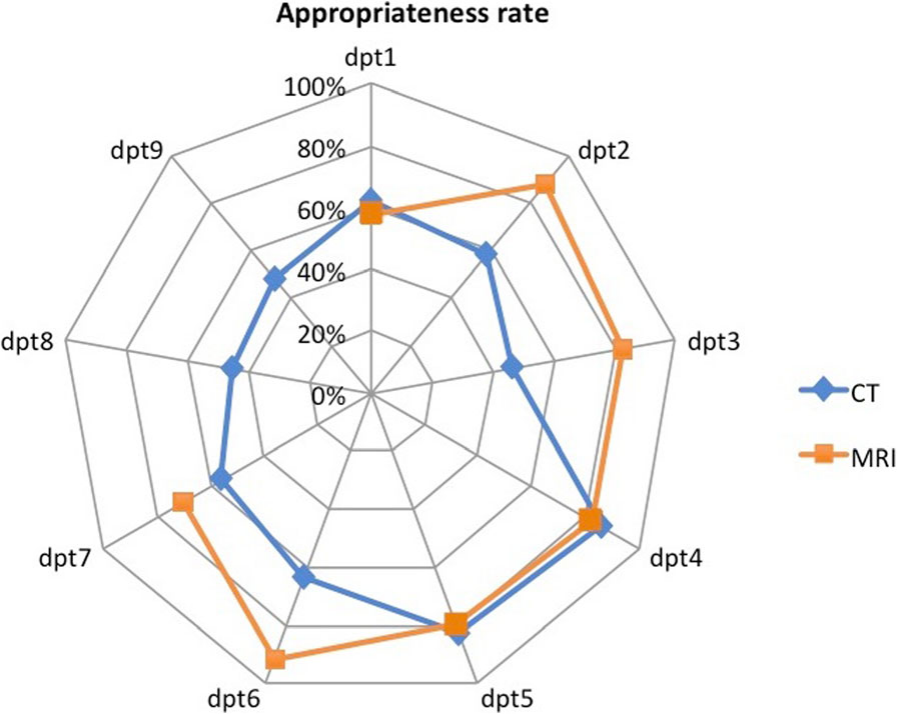
Radar chart showing AR for each radiology department (dpt) for CT and MRI requests

Figure 3 presents AR depending on the radiology department for both CT and MRI examinations. Huge variations of AR depending on the radiology department to which the request was addressed were observed for both CT requests (46–86%) and MRI requests (57–92%). AR for CT requests was higher (*p* = 0.004) for the seven radiology departments equipped with both CT and MRI units (65%) than for the two others (47%). The proportion of CT requests from medical specialists was also higher in the radiology departments equipped with MRI units (74%) than in the others (47%). Further comparison for each anatomical area revealed that AR for extremity CT was significantly higher (*p* = 0.002) for the radiology departments equipped with both CT and MRI units (21/32; 66%) than for the others (1/11; 9%), while no such variation was found for the other anatomical areas.

### Other findings concerning inappropriate requests

**Fig. 4:**
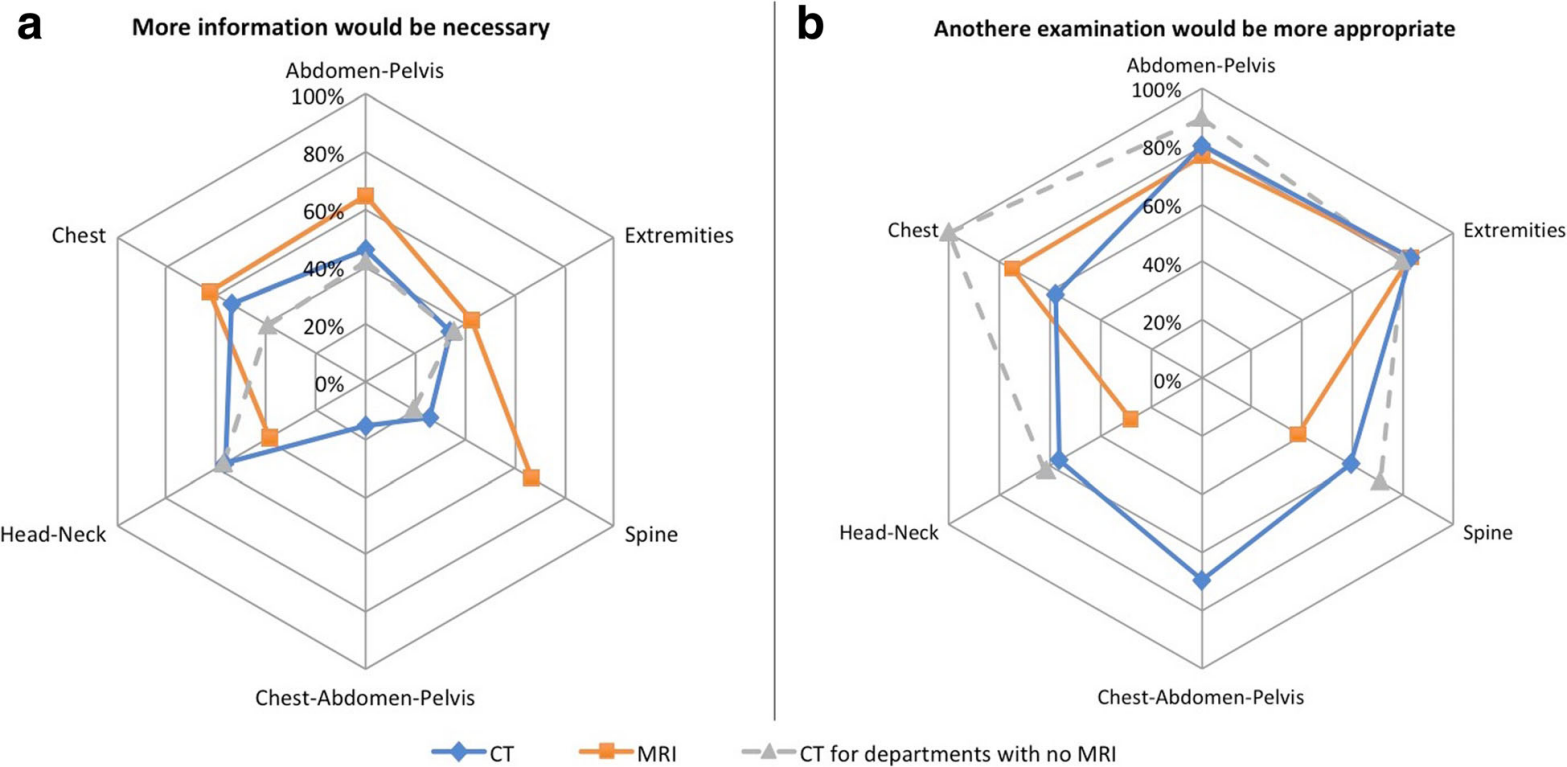
**a** Radar chart showing for each anatomical area the proportion of inappropriate requests for which more information would be necessary: for CT requests, for MRI requests and for CT requests for the two radiology departments not equipped with MRI units. **b** Radar chart showing for each anatomical area the proportion of inappropriate requests for which another examination would be more appropriate: for CT requests, for MRI requests and for CT requests for the two radiology departments equipped with only CT units

Analysis of the subsidiary questions concerning inappropriate requests revealed that the auditors needed more information in 35% of cases for CT (53/152) and 55% for MRI (38/69), and deemed another examination was more appropriate in 67% of cases for CT (102/152) and 58% for MRI (40/69). Figure 4 presents these proportions for each anatomical area for both CT and MRI requests. The auditors considered there was a lack of information for only 26% of cases for spinal CT requests (17/66).

In the case of inappropriate requests for which the auditors considered that another examination was more appropriate, instead of a CT examination they proposed MRI (47%), radiography (26%), ultrasound (22%), nuclear medicine (2%) or another type of imaging examination without ionising radiation (3%), whereas instead of MRI they proposed ultrasound (47%), radiography (35%), CT (11%), nuclear medicine (4%) or another type of examination without ionising radiation (3%). Further analysis of the inappropriate CT requests from the two radiology departments having no MRI units showed that another examination was deemed more appropriate in 78% of cases (60/77), and it was MRI in 55% of the cases.

**Fig. 5:**
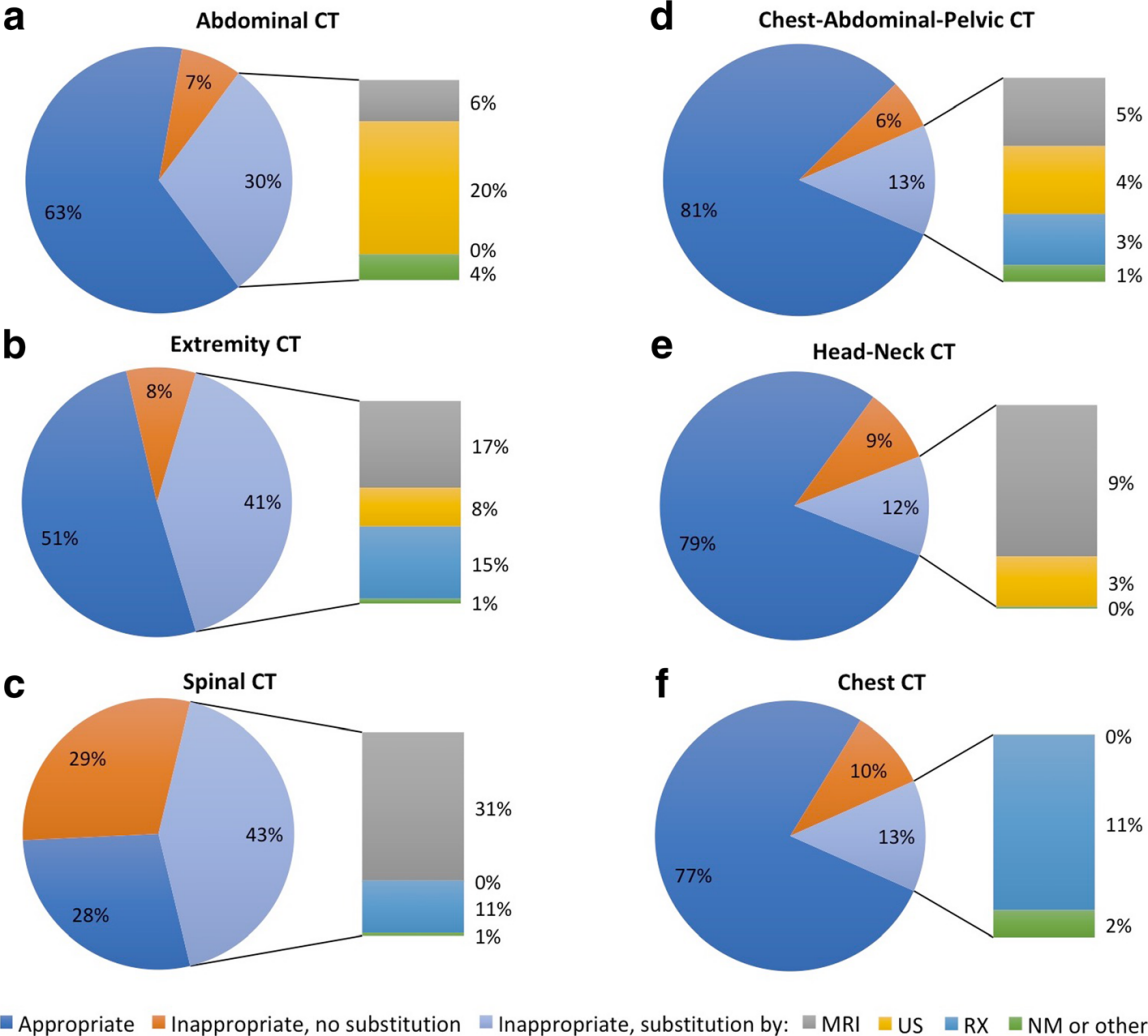
Diagrams showing for CT for each anatomical area (**a** abdomen; **b** extremities; **c** spine; **d** chest-abdomen-pelvis; **e** head-neck; **f** chest): the proportion of appropriate requests, the proportion of inappropriate requests for which no other examination was proposed instead and the proportion of different types of other examinations that were proposed. *CT* computed tomography, *MRI* magnetic resonance imaging, *US* ultrasonography, *RX* radiography, *NM* nuclear medicine, *other* another type of imaging examination without ionising radiation

Figure 5 shows, for CT for each anatomical area, the proportion of appropriate requests, the proportion of inappropriate requests for which no other examination was proposed instead and the proportion for which different types of other examinations were proposed. MRI was considered more appropriate than CT in 31% of cases for the spinal area (20/66) and in 17% of cases for the extremity area (4/21), whilst ultrasonography was considered more appropriate than CT in 20% of cases for the abdominal-pelvic area (6/28).

Further analysis concerning CT requests for the two radiology departments having no MRI unit revealed that MRI was considered more appropriate than CT in 42% of cases for the spinal area (8/18) and in 39% of cases for the extremity area (4/10).

### Presence and use of the recommendation in the guidelines

Overall, the auditors found in the guidelines the clinical situation that was described in the requests in 73% of cases for CT (283/388) and 79% for MRI (261/330). For CT, they found it for 78% of appropriate requests (183/236) and for 66% (100/152) of inappropriate requests. For MRI, the auditors found the clinical situation for 85% of appropriate requests (222/261) and for 57% of inappropriate requests (39/69).

**Fig. 6:**
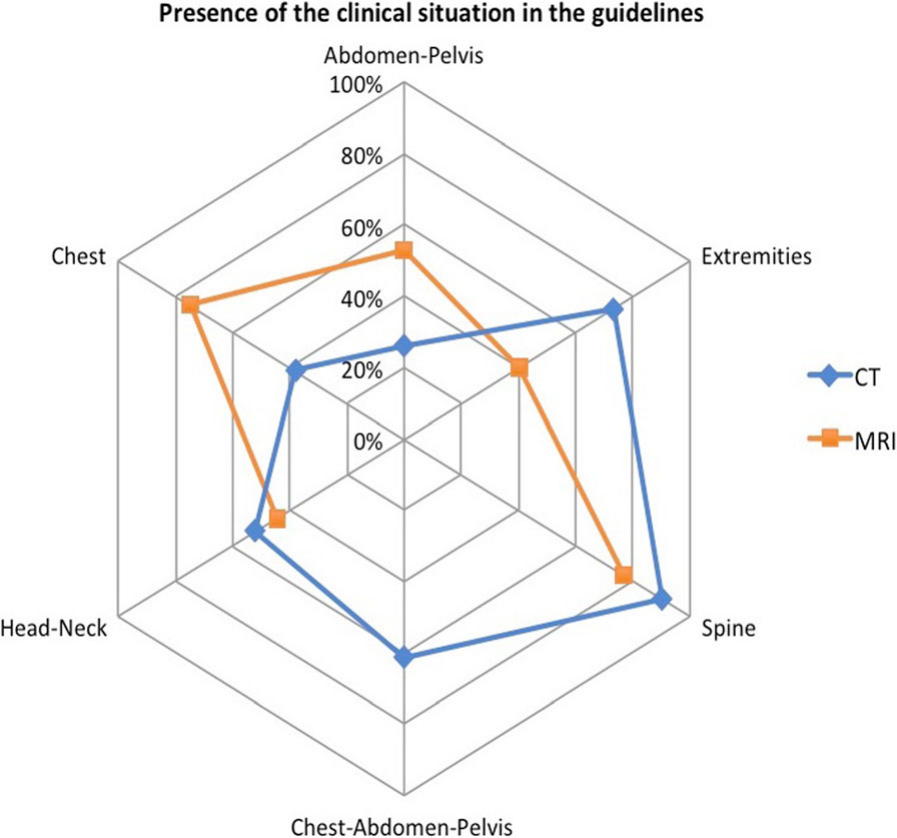
Radar chart showing for each anatomical area the proportion of inappropriate CT and MRI requests for which the described clinical situation was found in the referral guidelines

Figure 6 presents the proportions of inappropriate requests for which the clinical situation was found in the guidelines according to the anatomical area. This was the case for 90% of inappropriate spinal CT requests (59/66) and for 77% of inappropriate extremity CT requests (16/21).

The conclusion of the auditors was always in accordance with the recommendation of the guidelines except in the three following cases. Two requests for abdomen-pelvis CT for the assessment of lymph node and bone metastasis in patients with prostate cancer were not in accordance with the guidelines that recommended an MRI instead, but the auditors considered that this recommendation was ambiguous and that in fact these two requests were appropriate. Another request of chest CT for suspicion of pulmonary embolism in a child was in accordance with the recommendation in the guidelines, but the auditors considered that the recommendation was not suitable in this particular paediatric context and that the request was inappropriate.

## Discussion

Overall, the appropriateness of requests was unsatisfactory, especially for CT with 39% of inappropriate requests. Considering the fact that the audit concerned requests for already performed examinations, this figure raises high concerns regarding potential non-justified exposure of patients to ionising radiations.

The principle of justification applied to medical exposures makes it clear that if for a particular clinical situation there is a choice between a CT examination and a MRI examination, then the latter should be preferred since it is a non-ionising modality. The audit indicated that for 67% of inappropriate CT requests, another examination was better justified than CT, and that it would be MRI in 47% of the cases. The radiology departments equipped with no MRI unit showed a lower appropriateness rate of CT requests than the others, in particular concerning extremity CT, possibly due to the fact that some local referrers are less prone to direct their patients to an MRI equipment in another department and prefer to request a CT instead.

It was decided to audit both CT and MRI requests principally in a radiation protection perspective, with prior hypothesis that misuse of CT could be linked to a lack of availability of MRI in some cases, and this could in turn be linked to a misuse of MRI. The appropriateness rate was significantly higher for MRI requests (79%) than for CT (61%) requests, but also not satisfactory. The audit also revealed that for 58% of the inappropriate MRI requests, the MRI examination should be substituted by another type of examination, which could be ultrasound in most cases (47%).

Not surprisingly, requests for CT and MRI from medical specialists were more appropriate that those from general practitioners, the latter having by definition to deal with a broader spectrum of clinical situations. Our study also revealed better appropriateness for CT requests concerning children rather than adults, and that observation was comforted by the fact that each pair of auditors both included a radiologist specialised in paediatric imaging. More than 70% of the requests for spinal CT examinations were inappropriate, and most of them concerned lumbar spine CT. These results suggest that the focus for improvement should be on requests from general practitioners and on spinal CT examinations.

### Limitations

Potential sources of bias and uncertainties were introduced during the process of collection and rejection of requests. The radiology departments were simply asked to provide requests concerning consecutive examinations performed during a particular week, and this imperfectly randomised process may have induced potential bias in the distributions of the examinations that were collected by the RPD. The variation of results that we observed depending on the radiology department was probably linked in part to the variation of the type of examinations which are carried out in each department. Furthermore, 16% of the CT requests and 4% of the MRI requests initially collected were rejected from analysis because at least one of the auditors provided no conclusion concerning their appropriateness, in most of the cases because he considered that the clinical elements for justification were not comprehensive. Retrospectively, a better way to deal with these requests would have been to include them during the search of consensus with each pair of auditors, but it was not done because this situation had not been anticipated since a conclusion from each auditor was initially expected for all requests. However, neither the comparison between the distribution of the samples of requests before and after rejection nor the comparison between the proportion of rejected requests that only one auditor considered as appropriate and the AR values for the included requests revealed a substantial difference.

One major limitation in our study was the fact that the auditor evaluated the appropriateness of the request without any possibility of consulting other sources of information available such as previous medical records of the patients, communication with the medical referrer or consultation with the patient. The auditors indicated that not enough information was available in 35% of inappropriate CT requests and 55% of inappropriate MRI requests. Considering the fact that the radiologists working in a hospital could have the possibility to consult other sources of information in the process of individual justification of medical exposures, they would be able to justify an examination even if the request was inappropriate. Additional consultation of the patient files by the auditors in the methodology for evaluation could have presented the advantage of giving a clearer answer to the question of the appropriateness of the examination performed, but it was chosen not to proceed in this way because it would have been too complex and time consuming to realise in the context of a national audit with external auditors from other countries.

### Comparison with other studies

To our knowledge, only two other audits of imaging requests have already been performed on a national scale: one conducted in 2005 in Sweden [15] and the other in 2014 in the UK [16]. Our method was much closer to that employed in Sweden: the audit was also initiated by the national Radiation Protection Authority that engaged a number of physicians in order to individually assess the justification of 2435 CT requests of already performed examinations, based on both referral guidelines and their clinical experience. Overall, they found better results with only 20% of inappropriate CT requests, compared to 39% in our study. Interestingly, they also found variations depending on the anatomical area concerned by the requests, with similarly an appropriateness rate lower for spine (57%) than for other areas.

The methods and results from the UK national audit are not fully comparable with ours: it was an internal audit coordinated by a professional medical society and the 1890 CT and 1250 MRI requests were directly audited by radiologists working in the radiology department performing the examinations. Appropriateness rates were dramatically higher, with 93% for CT requests and 95% for MRI requests. Moreover, that audit only concerned requests provided by general practitioners, for which we report even lower appropriateness rates than overall (37% for CT; 64% for MRI). The audit realised in the UK also revealed that vetting of the referrals prior to examination was performed by radiologists in more than 95% of cases for both CT and MRI, something that is probably not performed that often in Luxembourg and could explain the better results observed in the UK.

In our study, we chose to contract external auditors from other countries in order to limit potential bias related to the conduct of internal audits. The design, results and scale of our audit, considering the number of radiology departments present in Luxembourg, are relatively much closer to those of a multicentre audit on the appropriateness, realised in Belgium in 2015 in eight radiology centres [17]. A review of 331 CT requests by multiple radiologists revealed an overall inappropriateness rate of 29%. They found the worst results for lumbar spine CT with 46.5% of inappropriate requests, a high proportion of which should have been substituted by a MRI examination.

Other audits of appropriateness of examinations realised on a smaller scale included additional consultation of the patient files. An internal audit performed at a university hospital in Finland revealed only 7% of inappropriate MRI examinations (10/150) [18]. Two other audits in the same hospital before and after specific interventions [19, 20] showed improvements with a decrease of inappropriate CT examinations in young adults and children from 29% in 2005 to 9% in 2009. The results according to the anatomical area in the first audit showed dramatically more inappropriate examinations for the lumbar spine area (77%) than for other anatomical areas, a great proportion of which should have been performed by an MRI instead. A recent cross-sectional study in Southern Italy [21] revealed that overall 79.4% of CT examinations (596/751) and 83.6% of MRI examinations (310/371) were appropriate, but that only 66.7% of MRI examinations (38/57) concerning spine and extremities were appropriate.

In our study, each request was evaluated by two auditors prior to a search for consensus. Prior inter-agreement between the two radiologists of each pair was “moderate”, which was worse than the substantial agreement between two radiologists reported with the same kappa test in the Belgian multicentre study, but probably more consistent than the “high inter-observer variance” between radiologist and clinician reported in the Swedish national survey. Meetings for reaching a consensus were not only necessary to consolidate our results, but they also provided the coordinator team with a great opportunity to be made aware of the practical difficulties encountered by the auditors in evaluating the requests, and of the practical daily challenges encountered in the radiology departments by radiologists who try to vet the requests before imaging. Prior disagreement of auditors appeared from discussions to be mainly due to a lack of sufficient information on the requests, leading to different interpretations when trying to find corresponding recommendations in the referral guidelines.

Our study is the first reported national audit of requests performed by radiologists from other countries. The neutrality of the auditors and lack of conflicts of interest were considered to be of importance for this audit.

### How to improve?

Concerning CT, in almost one-tenth of all cases, the auditors indicated that MRI would be more appropriate, and the appropriateness was better for the radiology departments equipped with both CT and MRI units than for the others. These results suggest that increasing the MRI capability may help to improve the justification of CT examinations. Nevertheless, this would probably not solve all the issues, considering the fact that the appropriateness is not excellent for MRI either.

The better results we observed for CT requests concerning children compared to adults suggest that other qualitative aspects such as risk awareness could also play a role. Some studies linked the inappropriate use of medical exposure to a low awareness of radiation dose and risk associated to medical imaging for both medical referrers and medical practitioners [22–24]. The implementation of a vetting process in the radiology departments could lead to improvement, as any feedback to referrers may contribute to active education and quality improvement [16].

In 2001, Luxembourg chose to adopt the French imaging referral guidelines, with the advantages of being available online for free and to be edited in French spelling. In about two-thirds of the requests they deemed inappropriate, the auditors indicated that they found the recommendation in the guidelines corresponding to the clinical situation described in the request, and their conclusions were in accordance with the recommendations in practically all cases. These results indicate that the guidelines should be helpful to avoid unnecessary exposures in a lot of situations, but also suggest that they are not sufficiently used or followed in Luxembourg. Education and training of general practitioners in the use of referral guidelines could potentially contribute to improvement, but one challenging particularity in Luxembourg is that the medical practitioners all gain a great part of their education and training in other European countries. Some studies point to a mitigated success of voluntary consultation of guidelines and then recommend the complementary use of Clinical Decision Support systems [25, 26], such as the i-Guide developed by ESR [27]. At the time of the study, no clinical decision systems were used in Luxembourg, but systems such as the ESR i-guide could be considered as soon as more feedback from pilot tests in other countries become available.

Conducting clinical audits could contribute to improving the quality of services provided by radiology departments, as the feedback they provide can contribute to the awareness of all relevant parties. Periodically repeating this kind of audit could be useful to monitor the evolution of AR in the long term, then potentially to evaluate the impact of future actions.

## Conclusions

Thanks to this audit, Luxembourg is aware that the appropriateness of CT and MRI is not acceptable and should be improved. Moreover, it indicates that the focus should be on general practitioners and on spine CT examinations. It can be hoped that following this audit, actions will be taken by all relevant parties in order to improve the situation.

## Additional file

Additional file 1: Instructions to auditors for carrying out the evaluation of the appropriateness of each request. (PDF 47 kb)

## Abbreviations

AR: Appropriateness rate
RPD: Radiation Protection Department
