# Supplementary material for: National audit on the appropriateness of CT and MRI examinations in Luxembourg
Source: Insights Imaging. 2019 May 20;10:54. doi: 10.1186/s13244-019-0731-9 (PMC6527721; doi:10.1186/s13244-019-0731-9)
Supplement: Supplementary file 1 — Instructions to auditors for carrying out the evaluation of the appropriateness of each request. (PDF 47 kb) [file 13244_2019_731_MOESM1_ESM.pdf]

Instructions to auditors for carrying out the evaluation of the appropriateness of each request

Please complete the dedicated excel table by answering the following question for all the requests of your attributed lot.

	Question	Expected data	Guidance
**Identification**	Reference number?	Number	Copy from the request.
**Examination**	Imaging Modality?	Text	Indicate the imaging modality requested by the request, if available.
	Type of examination?	Text	Indicate the type of examination requested by the request, if available.
**Patient**	Patient gender?	Male / Female	Copy from the request, if available.
	Patient age group?	Adult / Child	Copy from the request, if available.
**Referrer**	Medical specialty of the referrer?	Text	Copy from the request, if available.
**Clinical elements of justification**	Clinical background?	Text	Summarize in a few words the clinical background that is described in the request, if available.
	Question to be answered by the examination?	Text	Summarize in a few words the question to be answered by the examination that is raised in the request, if available.
**Recommendation in the clinical guidelines**	Is the clinical situation present in the guidelines?	YES / NO	Indicate YES if you find in the referral guidelines a clinical situation corresponding to the clinical elements for justification that are described in the request.
	Section of the guidelines?<br>*	Selection	Select from the drop-down menu the title of the section of the referral guidelines in which you found the clinical situation
	Clinical situation in the guidelines?<br>*	Selection	Select from the drop-down menu the title of the clinical situation you found in the referral guidelines
	Are the clinical elements for justification consistent with the recommendation in the guidelines?<br>*	YES / NO	According to the recommendation you found in the clinical guidelines and based on the available clinical elements for justification provided on the request, indicate:<br>- YES if you consider that it would be appropriate to perform the examination that is requested;<br>- NO if you consider that that it would be inappropriate to perform the examination that is requested.
**Conclusion of the auditor**	Is the request appropriate?	YES / NO	Based on the available elements of justification provided on the request, on the recommendation of the guidelines, and on your own expertise as a radiologist, indicate:<br>- YES if you consider that it would be appropriate to perform the examination that is requested;<br>- NO if you consider that that it would be inappropriate to perform the examination that is requested.
**Complementary question regarding the reason of inappropriateness**	Would more clinical elements for justification be necessary?<br>**	YES / NO	Indicate YES if you consider that more clinical elements for justification than those provided on the request would be necessary to potentially consider it as appropriate;<br>Otherwise indicate NO.
	Is there another examination that would be more appropriate?<br>**	YES / NO	Indicate YES if you consider that another type examination of examination would be more appropriate than the one that was requested;<br>Otherwise indicate NO.
	Which type of examination would be more appropriate?<br>***	Text	Indicate which type of examination you consider to be more appropriate than the examination that was requested.
	Additional remarks?<br>****	Text	Indicate any potential difficulties encountered during the evaluation of the requests or any information that could contribute to the quality of the audit.

An answer to each question is expected, except in the following cases:

- * Complete only if the answer to the question ”Is the clinical situation present in the guidelines?“ is “Yes”
- ** Complete only if the answer to the question ”Is the request appropriate?“ is “No”
- *** Complete only if the answer to the question” Is another type of examination more appropriate?“ is “Yes”
- **** Optional
